# Editorial: Advanced nanobiosensors for non-invasive disease diagnosis

**DOI:** 10.3389/fbioe.2023.1208679

**Published:** 2023-05-23

**Authors:** Wenbo Lu, Yupeng Wang, Xiaowei Cao

**Affiliations:** ^1^ Key Laboratory of Magnetic Molecules and Magnetic Information Materials, Ministry of Education, School of Chemistry and Material Science, Shanxi Normal University, Taiyuan, China; ^2^ Institute of Translational Medicine, Medical College, Yangzhou University, Yangzhou, China

**Keywords:** surface-enhanced Raman scattering, electrochemical sensor, analytical technique, clinical application, human health and disease

Previous studies have shown that biosensors are widely used to detect macromolecules in organisms (such as glucose, fat and so on), but have not been used on a large scale in clinical medicine. In recent years, the rise of nanotechnology has made great contributions to the early detection of diseases and bioimaging. Nanotechnology is based on the size characteristics of nanoparticles to develop a lot of diagnostic applications, and nano-biosensor is the combination of nanotechnology and traditional biosensors. Nanomaterials of different types, shapes and sizes are applied to biosensors to improve the sensitivity and accuracy of detection. Compared with traditional biosensors, nano-biosensors have better sensitivity and repeatability. The application of various nanotechnology and nanomaterials will also be more conducive to the miniaturization, portability and wearability of biosensors, so as to develop more and more advanced sensing modes in clinical medicine applications.

At the present stage, there exist convincing evidence showing that nano-biosensors have great potential in clinical medicine application, which is characterized by extraordinarily sensitive and efficient detection of some pathogenic factors, response to them and pathological analysis. Moreover, with the progress in the field of nanomaterials in recent years, modern biomedical sensors have brilliant abilities to detect various subtle changes in the human body more accurately by taking advantages of the unique characteristics of nanotechnology and nanomaterials, extremely improving the efficiency of disease treatment. It is no exaggeration to say that nanobiosensors have a promising and bright future in clinical medicine. However, every coin has two sides. Although nanobiosensors have many good advantages and show great potential in clinical medicine, it also has some shortcomings. For example, many outstanding problems of nano-toxicology limit their application in biological systems. Some nanosensors may affect cellular metabolism and homeostas, thereby altering the distribution of cellular molecules and making it difficult to distinguish sensor-induced artifacts from fundamental biological phenomena, among other problems. These are some necessary topics worth studying before the wide application of nano-biosensors in clinical medicine.

In order to acquire new knowledge in the field of clinical medicine, especially in the sustainable production of nano-biosensors, we have been committed to the research project “nano-biosensors in clinical medicine application research”. However, research topics include not only nano-biosensors, but also discoveries in the field of clinical medicine and pathological analysis. This Research Topic presents a series of original research articles describing sensitive detection methods for constructing nanobiosensors to detect many clinical diseases. This editorial contains five original research articles from leading research groups. Here, we would like to express our sincere thanks to the efforts and contributions of all the people involved in the research of this subject.

Between these five studies, they are interrelated and different from each other. In the field of clinical application, nano-biosensors of different sensitive materials are constructed to carry out sensitive detection of different biological macromolecules (such as genetic material, protein, endocrine disrupting chemicals) in the human body, so as to conduct an early treatment or therapeutic effect on some diseases and environmental substances related to human health.

In the first and second articles, two different nano-biosensors were constructed respectively to carry out sensitive detection of genetic material (DNA&RNA) in the human body, and carry out experiments for early screening of gastric cancer and treatment of cerebral hemorrhage, respectively. Both articles have produced excellent results, showing promise for a wide range of applications in clinical medicine.

**FIGURE 1:**
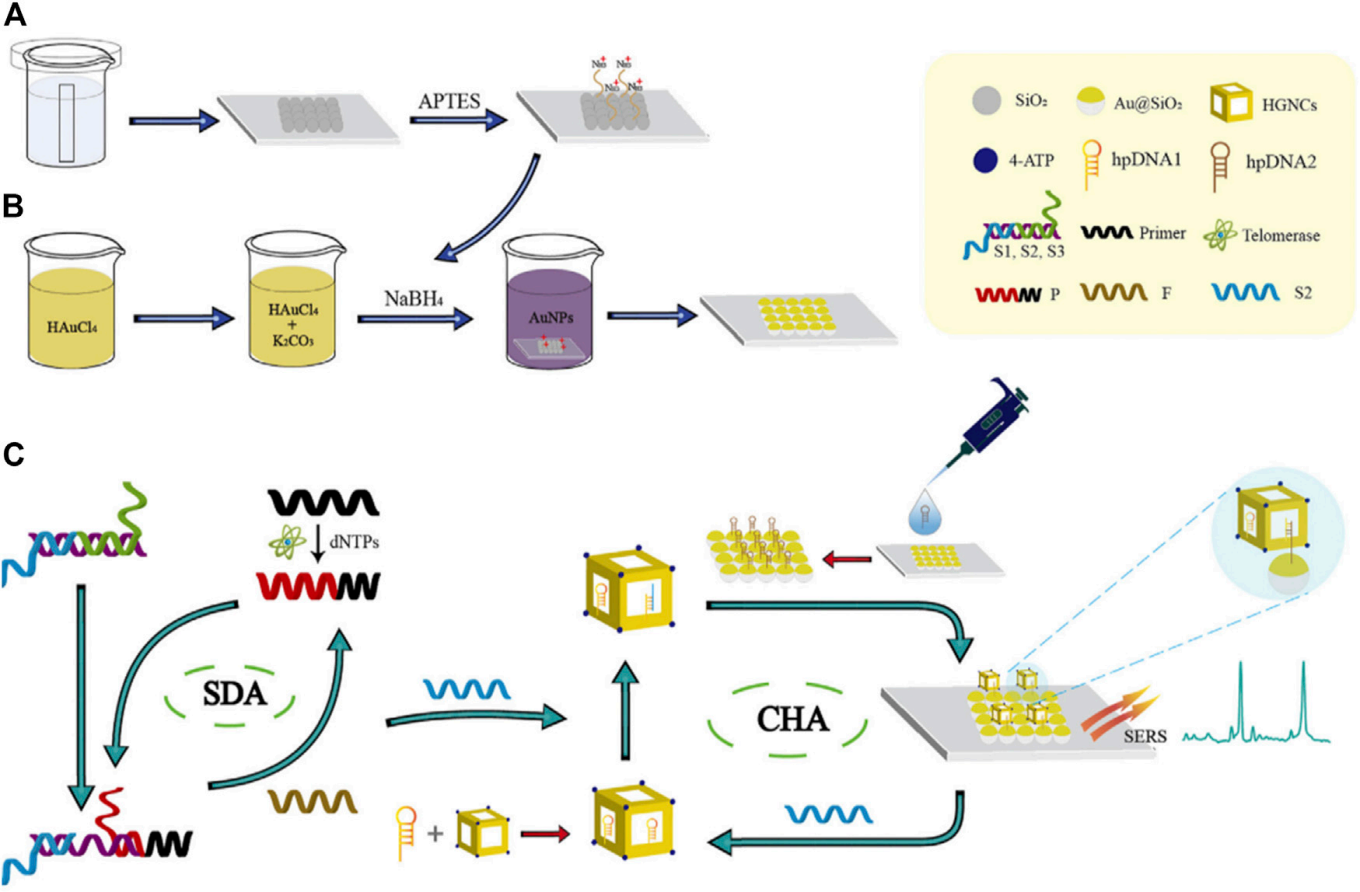
Schematic illustration of the biosensor combined with the SDA-CHA strategy for ultra-sensitive analysis of telomerase activity. **(A)** Synthesis of the single-layer SiO_2_ colloidal crystal film. **(B)** Fabrication of the Au@SiO_2_ array substrate. **(C)** The process of telomerase extension reaction and dual DNA-catalyzed amplification strategy.

The first paper is an original research paper by Shen et al. The novelty of this paper is that it describes the construction of a novel surface-enhanced Raman scattering biosensor to detect telomerase for early diagnosis and prognosis assessment of hepatocellular carcinoma using a double DNA catalytic amplification strategy assembled by chain shift amplification and catalytic hairpin (Shen et al.). As described in the figure, this work express the novel method that a single-layer SiO2 colloidal crystal film self-assembled by vertical evaporation was adsorbed by electrostatic adsorption on AuNPs in Figures 1A, B to prepare an ordered Au@SiO2 array. Hairpin DNA2 (hpDNA2) modified Au@SiO2 array, Raman molecular-labeled Au-AgNCs and hairpin DNA1 (hpDNA1) labeled Au-AgNCs were used as trapping substrate and SERS probes, respectively, as shown in Figure 1C. When telomerase and deoxyribonucleoside triphosphate (dNTPs) were present, telomerase primers could form long strand DNA containing repeated sequences (TTAGGG) to trigger SDA reaction. The product could initiate CHA reaction between SERS probes and captured substrates, attaching Au-AgNCs to the surface of Au@SiO2 array. As a result, local electromagnetic fields (or “hot spots”) would be significantly amplified, resulting in enhanced SERS signal. The biosensor constructed in this study could ideally be applied for early screening of liver cancer, which could have a wide range of applications in clinical medicine and is a method that deserves a vigorous promotion.

In the second paper, the sensitive detection of miRNA in the human body is achieved. A novel hydrophobic paper plasma SERS biosensor was fabricated by dropping DS gold nanorods of relatively narrow size and morphology onto H-paper (Wang Y. et al.). It plays an important role in the treatment, prognosis, or prevention of cerebral hemorrhage in patients. A range of experimental facts suggest that nanobiosensors have a wide range of applications in practical clinical detection.

The third and fourth articles focus on two novel methods of constructing nano-biosensors with the goal of specific detection of interleukin-6 in the human body, respectively, for clinical detection and treatment of human blood IL-6 content and subarachnoid hemorrhage disease, and show excellent performance.

This research paper originally explored the combination of 4-mercaptobenzoic acid and IL-6 antibody fixed on platinum carbon electrode modified with gold nanoparticles to construct an electrochemical sensor specifically recognizing IL-6, so as to detect interleukin specifically in the detection environment where many interfering proteins coexist (Wang C. et al.). The prepared nano-biosensor has been successfully applied in physical detection, and the content of IL-6 in biological samples has been detected, which shows excellent detection performance, and has a very broad application prospect.

Similarly, in the fourth paper, electrodes doped with gold nanoparticles were used for the same specific detection of interleukin-6 as in the previous paper to sensitively detect IL-6 levels in the blood of patients with subarachnoid hemorrhage.

It developed a novel free-labeled electrochemical immunosensor using AuNPs/THI (Au nanospheres) as an interfacial modified electrode to rapidly and sensitically detect IL-6 in the blood of subarachnoid hemorrhage (SAH) (Wang M. et al.). IL-6 in blood samples of patients with subarachnoid hemorrhage was detected by enzyme-linked immunosorbent assay (ELISA) and electrochemical enhancers. The constructed electrochemical immunosensor has been successfully implemented. The detection of target molecules in real samples shows great potential for clinical applications in subarachnoid hemorrhage.

Slightly different from the four articles mentioned above, the nano-biosensor constructed in the fifth article detects endocrine disrupting chemicals that are detrimental to the human body, which also shows promising development prospects in the field of clinical medicine for possible disease prevention.

Finally, an original research paper written by Shah et al. What will impress readers most is that it describes the progress and development of portable EDCs sensing technologies, including electrochemical, colorimetric, optical and microbial sensing methods for endocrine disrupting chemicals commonly found in daily life. It also describes the advantages and limitations of these sensing technologies and discuss the future development of sensing technologies for EDC environment sensing (Shah et al.).

Overall, the continued development of these sensors has the potential to provide a reference for understanding the exposure and health effects associated with these chemicals, and to provide substantive information for public health policies aimed at reducing health risks from exposure in a timely manner, which takes on the fierce practical significance.

Last but not least, in order to realize the application of nanomaterials in clinical medicine, a series of strategies are adopted to reduce the toxicity of nanomaterials, such as surface modification of nanomaterials to improve their biocompatibility, reducing the dose and exposure time of materials, adjusting the reaction environment of nanomaterials, and exploring degradable nanomaterials. However, there are still immerse amounts of challenges in this area of research. For example, the toxicity of most nanomaterials has not been fully addressed and can cause great damage to the bodies of experimentalists during the process of synthesis or use; Meanwhile, nanomaterials are not easy to degrade and can cause irreversible damage to the environment. Biosafety issues and environmental governance, as well as conservation, remain important factors limiting the clinical use of nanomaterials.

In conclusion, the articles published as part of the research theme “nanobiosensors in Clinical Medicine” provide excellent insights into the application of various metal-doped nanobiosensors in the detection of various clinical diseases. At the same time, it highlights some of the current challenges in the field of chemistry and clinical medicine, for example, the toxicity and harsh reaction environment of nanobiosensors, etc. We hope that this work will be successful in attracting other chemists to this exciting and rapidly developing field, and that more will be achieved in this area in the near future.

